# Fenugreek seed meal as a partial replacement for soybean meal: Dose-dependent effects on in vitro rumen fermentation and greenhouse gas production

**DOI:** 10.1038/s41598-026-61071-y

**Published:** 2026-08-10

**Authors:** Gouda A. Gouda, Gamal A. Mousa, Hossam H. Azzaz, Gamal M. El-Garhy, Asmaa A. Eid, Salah A. H. Abo El-Nor, Ahmed E. Kholif

**Affiliations:** 1 Dairy Science Department, National Research Centre, 33 Bohouth St. Dokki, Giza, Egypt; 2 Animal Production Department, Faculty of Agriculture, Fayoum University, Fayoum, 63514 Egypt

**Keywords:** Amino acids, Methane emissions, Nutrient degradability, Polyphenols, Protein meal, Rumen fermentation, Biochemistry, Biotechnology, Microbiology, Plant sciences

## Abstract

The use of alternative protein sources in ruminant diets has the potential to reduce feed costs and mitigate environmental impacts associated with conventional protein feeds. This study evaluated the effects of partially or completely replacing soybean meal (SBM) with graded levels of fenugreek seed meal (FSM; 0, 4.3, 8.6, 12.9, and 17.3% of DM) on in vitro ruminal fermentation, gas production, methane (CH_4_) and carbon dioxide (CO_2_) emissions, and nutrient degradability. FSM contained lower concentrations of essential amino acids (74.3 g/kg DM) than SBM (157 g/kg DM), whereas total polyphenols were higher in FSM (2014.9 µg/g) than SBM (1453.3 µg/g), particularly catechin, ellagic acid, methyl gallate, and rutin. Ruminal gas production kinetics were altered. The asymptotic gas volume (*b*) increased with 25% FSM inclusion (323.9 mL/g DM) relative to the control (300.7 mL/g DM; *P* = 0.005), but decreased at higher inclusion levels, with a significant quadratic effect (*P* = 0.014). The fractional gas production rate (*c*) and lag time were also affected (*P* ≤ 0.03), as was metabolizable energy, which peaked at FSM25 (*P* = 0.007). In vitro dry matter degradability and fiber degradation decreased linearly and quadratically at higher FSM levels (*P* < 0.05). Total volatile fatty acids, acetate, and propionate concentrations were higher at FSM25 compared to FSM0 but declined at greater FSM inclusion (*P* ≤ 0.037). Ruminal pH increased with higher FSM levels (*P* = 0.007). These results indicate that partial replacement of SBM with FSM (approximately 25% of SBM protein) can enhance ruminal fermentation and metabolizable energy, whereas higher inclusion rates reduce fiber degradability and alter fermentation end-products.

## Introduction

Ruminant livestock are essential to global food systems but contribute disproportionately to greenhouse gas (GHG) emissions, particularly methane (CH_4_) and carbon dioxide (CO_2_) from enteric fermentation, which are key drivers of climate change and represent an energy loss to the animal^1^. Enteric CH_4_ alone accounts for a significant share of livestock-related GHGs, prompting urgent research into dietary strategies that mitigate emissions without compromising animal performance. Dietary manipulation is recognized as one of the most feasible approaches to reduce enteric CH_4_, with plant-derived feedstuffs and byproducts increasingly investigated for their potential to alter rumen fermentation and lower GHG output^2^.

High costs associated with conventional protein feeds such as soybean meal (SBM), combined with environmental pressures related to land use, deforestation, and nutrient runoff, have driven interest in alternative protein sources to improve the sustainability and resilience of livestock production systems. Substituting SBM with locally available, lower-cost ingredients such as sesame meal^3^, wheat germ meal^4^, *Moringa oleifera*^5^, sunflower meal^6^, and *Nigella sativa*^7^ has shown promise in ruminant diets as alternative protein sources capable of supporting animal nutrition while reducing dependence on conventional protein supplements. Such substitutions may contribute to improved resource utilization and sustainability depending on ingredient availability, market conditions, and animal responses under practical production systems, while lessening environmental impacts.

Fenugreek (*Trigonella foenum-graecum* L.) seed meal (FSM) is one such alternative with emerging interest as a protein feed ingredient. Native to the Mediterranean Basin and the Indian subcontinent, fenugreek is a legume capable of thriving under arid conditions, making it a resilient crop in regions prone to heat and water scarcity^8^. Its seeds contain substantial carbohydrate (~ 58%), high-quality protein (~ 23–26%), fiber (~ 25%), and volatile oil (~ 6%), making them nutritionally dense compared to many conventional byproducts^9^. The quality of FSM protein has been reported to compare favorably with some alternative plant protein sources, particularly with respect to selected amino acids such as lysine when compared with cottonseed meal^9^. However, amino acid composition varies among fenugreek products and processing methods, and available information indicates that FSM generally contains lower concentrations of several essential amino acids than soybean meal. Therefore, its suitability as a protein source should be evaluated not only on crude protein concentration but also on amino acid composition and overall dietary context.

Beyond basic nutrient composition, fenugreek seeds are rich in secondary metabolites such as saponins, polyphenols, flavonoids, alkaloids, and other bioactive compounds^10^. Consequently, the value of FSM as a feed ingredient may derive not only from its protein contribution but also from its phytochemical composition, which can influence ruminal fermentation and methane production. These phytochemicals have demonstrated effects on ruminal microbes and fermentation pathways, including modulation of methanogens and protozoa, which may influence CH_4_ production and feed digestion^11^. Indeed, certain bioactive components in fenugreek have been linked with reduced CH_4_ output in vitro, likely due to impacts on hydrogen utilization and ruminal microbial communities^9^.

Despite these promising attributes, and despite the extensive literature describing the nutritional and phytogenic properties of fenugreek, important knowledge gaps remain regarding its application as a direct substitute for soybean meal (SBM) in ruminant diets. Most previous studies have evaluated fenugreek seeds, extracts, sprouts, or whole plants as feed additives, whereas relatively few have investigated fenugreek seed meal (FSM) as a protein source replacement. Moreover, available studies have often focused on animal performance, rumen fermentation, or methane mitigation at single or limited inclusion levels, making it difficult to establish dose-response relationships and identify optimal substitution rates. Some studies have shown improved fermentation and nutrient utilization with FSM or fenugreek extracts, while others report minimal effects on rumen parameters and CH_4_ output depending on species, form of fenugreek, or inclusion level^9,11^. This inconsistency suggests that responses to fenugreek may depend on both the dietary context and the protein source being replaced. Mousa et al.^11^ replaced cottonseed meal with FSM at 0, 50, 75, and 100% of the protein source and reported a 30–41% reduction in in vitro CH_4_ production along with decreased nutrient degradability. They also reported that substituting 50% of cottonseed meal with FSM optimized growth performance, improved health indicators, and enhanced economic returns in fattening lambs. Whereas FSM has been studied as a replacement for cottonseed meal^11^, its evaluation as a replacement for SBM, the globally dominant ruminant protein supplement, under systematic, graded dose-response conditions in an in vitro fermentation model is lacking. Given the compositional differences between SBM and FSM in amino acid profile, polyphenol content, and fiber fractions, and given that SBM is the primary reference protein source against which alternative proteins are evaluated in most ruminant nutrition systems, an FSM-vs-SBM comparison is both distinct from and more practically relevant than FSM-vs-cottonseed meal comparisons.

In light of the need to enhance ruminant nutrition while mitigating the environmental impact of livestock production, this study evaluated the effects of partially or completely replacing SBM with graded levels of FSM on in vitro ruminal fermentation. Key parameters included total gas production, CH_4_ and carbon dioxide (CO_2_) emissions, and fiber and dry matter (DM) degradability (*d*DM). The objective was not simply to confirm previously reported effects of fenugreek, but to determine the response pattern associated with progressive replacement of SBM by FSM and to identify whether the distinct nutritional and phytochemical characteristics of FSM influence ruminal fermentation differently from a conventional SBM-based diet. This work aimed to elucidate how FSM affects rumen fermentation dynamics and GHG production (mainly CH_4_ and CO_2_), providing insight into its potential as a sustainable protein alternative. Specifically, although previous studies have evaluated fenugreek seeds, extracts, or whole plants in ruminant diets, and one study replaced cottonseed meal with FSM at graded levels^11^, no study has comprehensively evaluated the dose-dependent replacement of SBM with FSM while simultaneously characterizing the nutritional, amino acid, and polyphenolic composition of FSM and relating these characteristics to fermentation responses and greenhouse gas emissions. The present study addresses this gap. We hypothesize that the distinctive nutrient composition of FSM, with its unique phenol profile (catechins, ellagic acid, methyl gallate, and rutin) and differences in fiber and amino acid composition relative to SBM differentially influence ruminal fermentation relative to conventional SBM. Therefore, graded inclusion of FSM may simultaneously improve feed utilization and reduce GHG emissions, supporting its application as an environmentally sustainable protein source in ruminant diets.

## Materials and methods

### Experimental protocol

All experimental procedures were reviewed and approved by the Fayoum University Institutional Animal Care and Use Committee (FU-IACUC) (Ethical Approval No. AEC 2516-a, April 6, 2025), and animal management complied with the Guide for the Care and Use of Agricultural Animals in Research and Teaching (3rd ed., Federation of Animal Science Societies, 2010). Rumen fluid was collected postmortem at a commercial slaughterhouse in accordance with local animal welfare and slaughter regulations; this collection procedure was covered under the same institutional approval and did not require a separate ethics review.

### Ingredients, treatments, and chemical characterization

A basal total mixed ration (TMR) was formulated to serve as the substrate for in vitro incubation. The TMR consisted (g/kg DM) of 500 g concentrate feed mixture, 300 g berseem clover (*Trifolium alexandrinum*), and 200 g wheat straw. Soybean meal in the concentrate portion was partially or totally replaced with FSM to generate five experimental diets. The control diet (FSM0) contained SBM as the sole protein source. In the remaining treatments, SBM was replaced with FSM at 25% (FSM25), 50% (FSM50), 75% (FSM75), or 100% (FSM100). The experimental diets were not formulated to be isonitrogenous, isoenergetic, or isofibrous; the replacement was implemented on a weight-for-weight basis in the concentrate fraction to reflect a practical substitution scenario.

The fenugreek seed meal expeller cake used in this study was obtained by mechanical cold-press extraction of oil from dried mature seeds of *Trigonella foenum-graecum* L., produced in a single commercial batch at Al Jasmine Factory for Natural Oils (Fayoum Governorate, Egypt). Following oil extraction, the meal was stored in sealed polyethylene bags under dry, ventilated conditions at ambient temperature (22–25 °C), protected from direct sunlight, and used within four weeks of production. Residual ether extract content of the FSM was 40.1 g/kg DM (Table 1), indicating that the material was partially defatted following oil extraction. This material differs from whole fenugreek seed flour in that it has a reduced residual fat content (40.1 g/kg DM; Table 1), a potentially altered bioactive compound profile due to mechanical pressing, and a different energy density. Results from this study therefore apply specifically to cold-pressed FSM and should not be extrapolated to whole seed or solvent-extracted meal. Prior to analysis, the FSM was hammer-milled to a particle size visually comparable to that of soybean meal (approximately 2–4 mm), although particle size distribution was not formally determined. Saponins, condensed tannins, hydrolysable tannins, and mycotoxins were not quantified in the present study and therefore their potential contribution to the observed fermentation responses could not be directly evaluated.

**Table 1:** Ingredients and chemical composition of feeding stuffs and total mixed rations of fenugreek seed meal trial.

Item	Diet^1^					Ingredients					
	FSM0	FSM25	FSM50	FSM75	FSM100	Berseem clover	Wheat straw	Yellow corn	Wheat bran	SBM	FSM
Ingredients (g/kg DM)											
Berseem clover	300	300	300	300	300						
Wheat straw	200	200	200	200	200						
Yellow corn	275	275	275	275	275						
Wheat bran	95	80.6	66.25	51.85	37.5						
Soybean meal	115	86.25	57.5	28.75	0						
Fenugreek seed meal	0	43.15	86.25	129.4	172.5						
Limestone	7.5	7.5	7.5	7.5	7.5						
Premix	2.5	2.5	2.5	2.5	2.5						
NaCl	2.5	2.5	2.5	2.5	2.5						
NaHCO_3_	2.0	2.0	2.0	2.0	2.0						
Antitoxin	0.5	0.5	0.5	0.5	0.5						
Chemical composition (g/kg)											
OM	881.3	879.8	878.4	876.9	875.4	861	839	948	904.4	944.3	896.7
CP	132.5	130.0	127.5	125.0	122.5	144.5	19.0	84.0	138.5	427.0	273.0
EE	25.1	25.7	26.4	27.1	27.8	37.7	7.7	25.3	28.1	22.5	40.1
NSC	393.4	392.7	391.9	391.1	390.3	286.8	81.3	757.7	423.6	369.8	369.6
Ash	118.7	120.2	121.6	123.1	124.6	139.0	161.0	52.0	95.6	55.7	103.3
NDF	330.3	331.4	332.5	333.7	334.8	392.0	731.0	81.0	314.2	125.0	214.0
ADF	187.5	187.6	187.6	187.6	187.6	175.0	543.0	17.4	125.8	84.4	98.8
Hemicellulose	142.8	143.8	144.9	146.1	147.2	217.0	188.0	63.6	188.4	40.6	115.2
GE (MJ/kg DM)^2^	16.5	16.4	16.4	16.4	16.3	16.0	15.3	17.3	17.1	19.4	17.9

^1^The control diet based on (per kg DM): 500 g of concentrate feed mixture, 300 g berseem clover (*Trifolium alexandrinum*), and 200 g wheat straw (FSM0 diet). Fenugreek seed meal (FSM) was included at 4.3%, 8.6%, 12.9% and 17.3% to replace soybean meal at 25% (FSM25 diet), 50% (FSM50 diet), 75% (FSM75 diet) or 100% (FSM100 diet), respectively. ADF, Acid detergent fiber; CP, Crude protein; EE, Ether extract; NDF, Neutral detergent fiber; NSC, Non-structural carbohydrates; OM, organic matter; GE, gross energy. ^2^Calculated according to MAFF^49^.

Polyphenol concentrations in SBM and FSM were determined using an Agilent 1260 Infinity HPLC System (Agilent Technologies, Santa Clara, CA, USA) equipped with an Eclipse C18 column (4.6 mm × 250 mm i.d., 5 μm). The mobile phase consisted of water (A) and 0.05% trifluoroacetic acid in acetonitrile (B) at a flow rate of 0.9 mL/min. The gradient program was as follows: 0 min (82% A); 0–5 min (80% A); 5–8 min (60% A); 8–12 min (60% A); 12–15 min (82% A); and 15–20 min (82% A). Detection was carried out at 280 nm using a multi-wavelength detector. The injection volume was 5 µL and column temperature was maintained at 40 °C. External standards (Sigma-Aldrich GmbH, Steinheim, Germany) were used for identification and quantification of phenolic compounds.

Amino acid profiles of SBM and FSM were analyzed using the same HPLC system following acid hydrolysis as described by Jajic et al.^12^. Briefly, 0.1 g of sample was mixed with 2.5 mL distilled water and 2.5 mL of 6 M HCl, sealed, and hydrolyzed at 100 °C for 24 h. After filtration, 1 mL of filtrate was evaporated to dryness and reconstituted in 0.1 M HCl prior to injection. Separation was achieved using an Eclipse Plus C18 column (4.6 mm × 250 mm, 5 μm). The mobile phase consisted of sodium phosphate dibasic and sodium borate buffer (pH 8.2) as solvent A and ACN:MeOH: H_2_O (45:45:10) as solvent B at a flow rate of 1.5 mL/min. Detection was performed using a diode array detector at 338 nm (bandwidth 10 nm) and a fluorescence detector set at 340/450 nm (excitation/emission) from 0 to 27 min and 266/306 nm from 27 to 35 min. Amino acid standards (Sigma, Product #A6282, MO, USA) were used for calibration.

### In vitro fermentation and gas production

The in vitro fermentation medium was prepared according to Goering and Van Soest^13^. A reducing solution containing sodium sulfide (2 mL) was added to the buffer immediately before inoculation. Buffered rumen fluid was prepared by mixing ruminal inoculum (20 mL) with buffer solution (80 mL) in 250 mL bottles under continuous CO_2_ flushing to maintain anaerobic conditions.

Ruminal inoculum was collected from three Barki sheep at a local slaughterhouse. Prior to slaughter, animals were fed ad libitum a diet consisting (DM basis) of concentrates, berseem hay, and rice straw at a ratio of 500:400:100, with free access to water. Feed was offered at approximately 07:00 h; slaughter was conducted at approximately 10:00 h (approximately 3 h post-feeding), ensuring the rumen was in an active fermentation state. Slaughter procedures complied with animal welfare regulations. Rumen contents were collected within 10 min postmortem following the standardized procedure described by Fortina et al.^14^. Approximately 150–250 g of rumen contents were manually squeezed through a colander into a plastic beaker, and the process was repeated until approximately 1000 mL of rumen fluid was obtained. The collected fluid was filtered through two layers of cheesecloth to remove large feed particles, and the retained solids were squeezed to recover adherent microorganisms. The initial pH of the inoculum ranged from 6.8 to 6.9.

Approximately 1.0 g (± 10 mg) of each TMR sample (DM basis) was weighed into ANKOM F57 filter bags (Ankom Technology, Macedon, NY, USA) and transferred to 250 mL fermentation bottles connected to the ANKOM RF Gas Production System equipped with an automatic wireless in vitro gas production module and integrated pressure sensors (Ankom Technology, Macedon, NY, USA). Headspace pressure was recorded at 10-min intervals over a 48-h incubation period, and cumulative pressure values were calculated accordingly. Recorded pressure data were converted to gas volume (mL) under standard temperature and pressure conditions. Net gas production was obtained by correcting total gas volume for blank bottles containing inoculum without substrate. After 48 h of incubation, 5 mL gas samples were withdrawn from the sampling port and analyzed for CH_4_ and CO_2_ concentrations using a Gas-Pro detector (CROWCON Model Tetra3, Abingdon, UK). The detector was calibrated prior to use against certified reference gas mixtures (20% CH_4_, 20% CO_2_, balance N_2_) traceable to national standards. Detection limits are 100 ppm for CH_4_ and 200 ppm for CO_2_, with measurement repeatability (%CV) of less than 3%. Gas composition analysis was performed at the 48-h incubation endpoint only, as repeated headspace sampling at multiple time points would have released accumulated pressure and disturbed the anaerobic conditions of the closed fermentation vessels. The single-endpoint measurement therefore reflects the cumulative composition of fermentation gases rather than a time-course profile. Each treatment was incubated in three separate runs with three replicates per run. In each run, three blank bottles were included to correct for background fermentation.

After 48 h of incubation, fermentation was terminated by placing the bottles on ice for 5 min. The pH of the fermentation medium was immediately measured using a calibrated pH meter. It should be noted that ANKOM F57 filter bags have a nominal pore size of approximately 25 micrometers, which may have restricted physical access by larger ruminal microorganisms such as ciliated protozoa (typically 20–200 micrometers in diameter), potentially reducing protozoa-associated methanogenesis and affecting comparability with open-bottle fermentation systems. Filter bags were removed, rinsed, and dried in a forced-air oven at 55 °C for 48 h to determine dDM. Neutral detergent fiber (NDF) and acid detergent fiber (ADF) disappearance were determined by subtracting the weight of the dried residue from the initial substrate weight. Methane and CO_2_ production were expressed as a percentage of total gas and relative to incubated DM (mL/g DM), digested DM (mL/g *d*DM), digested NDF (mL/g *d*NDF), and digested ADF (mL/g *d*ADF).

Approximately 5 mL of fermentation supernatant was collected for determination of ammonia-N (NH_3_–N) and volatile fatty acids (VFA). For NH_3_–N analysis according to AOAC^15^, 3 mL of supernatant was mixed with 3 mL of 0.2 M HCl. For VFA determination, 0.8 mL of the supernatant from each bottle was mixed with 0.2 mL of metaphosphoric acid (250 g/L) and prepared for chromatographic analysis. Individual VFAs including acetate (C_2_), propionate (C_3_) and butyrate (C_4_) were quantified by high-performance liquid chromatography (HPLC). Separation was achieved using an Eclipse AQ-C18 HP column (4.6 × 150 mm i.d., 3 μm particle size), with 0.005 N H_2_SO_4_ as the mobile phase. The flow rate was programmed in a linear gradient as follows: 0–4.5 min at 0.8 mL/min; 4.5–4.7 min at 1.0 mL/min; 4.7–4.71 min at 1.0 mL/min; 4.71–8.8 min at 1.2 mL/min; 8.8–9.0 min at 1.3 mL/min; 9–23 min at 1.3 mL/min; and 23–25 min at 0.8 mL/min. Detection was performed using a diode array detector set at 210 nm, with an injection volume of 5 µL per sample and a column temperature maintained at 55 °C. External calibration was conducted using a standard mixture of individual VFAs (Sigma Chemie GmbH, Steinheim, Germany). All analyses were carried out at the Chromatography Laboratory, Central Laboratories Network, National Research Centre, Egypt.

### Chemical analysis

Dry matter, ash, crude protein (CP), and ether extract were determined according to AOAC^15^ procedures. Neutral detergent fiber was analyzed using sodium sulfite with α-amylase following Van Soest et al.^16^. Acid detergent fiber was determined according to AOAC^15^ and expressed exclusive of residual ash. Organic matter, hemicellulose, and non-structural carbohydrates (NSC) were calculated accordingly.

### Calculations and statistical analysis

Cumulative gas production data (mL/g DM) were fitted to the nonlinear model of France et al.^17^ using the NLIN procedure of SAS (Version 9.4, SAS Institute Inc., Cary, NC, USA): y = b × [1 − e^− c (t−Lag)^]; where *y* is the volume of gas produced at time *t* (h), *b* is the asymptotic gas production (mL/g DM), *c* is the fractional rate constant (/h), and *Lag* is the discrete lag time (h).

The partitioning factor at 48 h (PF_48_; mg dDM/mL gas) was estimated according to Blümmel et al.^18^. Gas yield at 24 h (GY_24_; mL/200 mg DM) and metabolizable energy (ME) were calculated according to Menke et al.^19^.

All statistical analyses were performed using SAS software (9.4, SAS Institute Inc., Cary, NC, USA). Data were analyzed using the PROC MIXED procedure. The experimental unit was the individual fermentation flask. Run was included as a random effect in the PROC MIXED model to partition run-to-run inoculum variation from residual flask-level error. Treatment means were separated using the PDIFF option with Tukey–Kramer adjustment to control the family-wise error rate. The statistical model was: Y_ijk_ = µ + T_i_ + R_k_ + ε_ijk_, where Y_ijk_ represents the observation for the *j*th flask in the *i*th treatment within the *k*th run, µ is the overall mean, T_i_ is the fixed effect of dietary treatment (FSM0 to FSM100), R_k_ is the random effect of run (k = 1 to 3; R_k_ ~ N(0, σ^2^r)), and ε_ijk_ is the residual error term. When appropriate, orthogonal polynomial contrasts were used to assess linear and quadratic responses to increasing levels of FSM replacing SBM. Differences among treatment means were considered statistically significant at *P* < 0.05.

## Results

### Chemical composition

The inclusion of FSM in the diets led to predictable changes in chemical composition due to the replacement of SBM (Table 1). As FSM increased from 0% (FSM0) to 17.3% (FSM100), CP content decreased from 132.5 g/kg DM to 122.5 g/kg DM. Ether extract increased slightly from 25.1 to 27.8 g/kg DM. Fiber fractions also increased with FSM inclusion, with NDF from 330.3 to 334.8 g/kg DM, and hemicellulose from 142.8 to 147.2 g/kg DM, while ADF remained relatively constant at ~ 187.6 g/kg DM. Non-structural carbohydrates decreased progressively from 393.4 g/kg DM (FSM0) to 390.3 g/kg DM (FSM100). Gross energy slightly declined from 16.5 to 16.3 MJ/kg DM with increasing FSM inclusion.

The amino acid profiles of SBM and FSM are presented in Table 2. SBM contained higher concentrations of essential amino acids than FSM, particularly leucine (30.9 vs. 10.3 g/kg DM), isoleucine (16.5 vs. 6.1 g/kg DM), and valine (15.9 vs. 5.9 g/kg DM). Lysine content, which is critical for ruminant growth, was 12.1 g/kg DM in SBM compared to 7.9 g/kg DM in FSM. Among nonessential amino acids, glutamic acid was predominant, with SBM containing 69.4 g/kg DM versus 29.2 g/kg DM in FSM. Arginine was relatively high in FSM (17.1 g/kg DM) compared to other nonessential amino acids. Total essential amino acids were substantially higher in SBM (157 g/kg DM) than FSM (74.3 g/kg DM), while total nonessential amino acids were 171.1 and 67.6 g/kg DM for SBM and FSM, respectively. Cystine and proline were not detected in FSM.

**Table 2:** Amino acids profile of soybean meal (SBM), and fenugreek seed meal (FSM) (g/kg DM).

Amino acid	SBM (mean ± SE)	FSM (mean ± SE)
Histidine	9.8 ± 0.4	4.8 ± 0.16
Methionine	5.8 ± 0.19	2.8 ± 0.13
Threonine	14.0 ± 0.35	6.5 ± 0.27
Valine	15.9 ± 0.31	5.9 ± 0.25
Lysine	12.1 ± 0.26	7.9 ± 0.38
Leucine	30.9 ± 0.68	10.3 ± 0.54
Isoleucine	16.5 ± 0.33	6.1 ± 0.26
Phenylalanine	17.6 ± 0.37	7.2 ± 0.27
Glutamic acid	69.4 ± 1.44	29.2 ± 1.06
Aspartic acid	38.0 ± 0.82	14.8 ± 0.47
Glycine	15.5 ± 0.34	8.3 ± 0.22
Serine	16.7 ± 0.37	8.1 ± 0.27
Alanine	15.0 ± 0.38	7.2 ± 0.25
Arginine	24.2 ± 0.53	17.1 ± 0.57
Tyrosine	10.2 ± 0.22	5.7 ± 0.23
Proline	14.7 ± 0.30	ND
Cystine	1.8 ± 0.051	ND
Σ essential amino acid	157.0 ± 2.47	74.3 ± 1.59
Σ nonessential amino acid	171.1 ± 4.19	67.6 ± 2.77

ND , not detected.

Polyphenol concentrations differed markedly between SBM and FSM (Table 3). FSM was particularly rich in catechin (977.5 µg/g) and ellagic acid (170.0 µg/g), while SBM contained higher chlorogenic acid (305.8 µg/g) and vanillin (252.5 µg/g). Total polyphenols were higher in FSM (2014.9 µg/g) than SBM (1453.3 µg/g). Other notable compounds in FSM included methyl gallate (165.6 µg/g), rutin (167.7 µg/g), and coumaric acid (32.6 µg/g), which were either absent or present at much lower levels in SBM. Conversely, ferulic acid and naringenin were higher in SBM than FSM.

**Table 3:** Concentration^1^ (µg/g) of polyphenols of soybean meal (SBM), and fenugreek seed meal (FSM) identified by HPLC analysis.

Compound	SBM (mean ± SE)	FSM (mean ± SE)
Gallic acid	54.1 ± 2.4	102.7 ± 5.44
Chlorogenic acid	305.8 ± 10.52	67.4 ± 3.54
Catechin	50.3 ± 2.31	977.5 ± 36.37
Methyl gallate	2.7 ± 0.11	165.6 ± 7.62
Caffeic acid	292.2 ± 10.35	198.0 ± 8.48
Syringic acid	21.4 ± 0.87	19.3 ± 1.38
Rutin	4.7 ± 0.24	167.7 ± 7.56
Ellagic acid	30.5 ± 1.42	170.0 ± 7.40
Coumaric acid	ND	32.6 ± 2.27
Vanillin	252.5 ± 9.53	16.4 ± 1.65
Ferulic acid	16.6 ± 0.67	11.5 ± 1.45
Naringenin	7.5 ± 0.35	2.0 ± 1.17
Rosmarinic acid	99.9 ± 3.55	25.8 ± 2.80
Daidzein	85.7 ± 3.02	14.7 ± 1.63
Quercetin	13.7 ± 0.58	38.3 ± 2.54
Cinnamic acid	ND	0.8 ± 0.062
Kaempferol	215.7 ± 7.59	4.6 ± 0.47
Σ polyphenols	1453.3 ± 58	2014.9 ± 85

^1^Concentration based on the total areas of the identified peaks.

^2^Identification based on authentic standards, the National Institute of Standards and Technology (NIST) library spectra, and literature.

ND, not detected.

### In vitro gas production kinetics

**Fig. 1:**
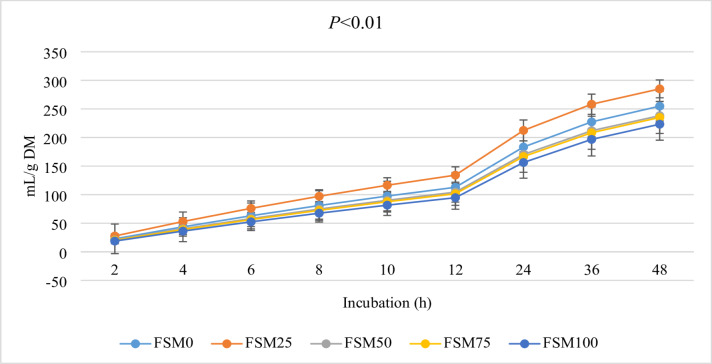
In vitro ruminal gas production (mL/g incubated DM) of diets containing fenugreek seed meal (FSM) at 0, 4.3%, 8.6%, 12.9%, and 17.3% of DM, replacing soybean meal at 0% (FSM0), 25% (FSM25), 50% (FSM50), 75% (FSM75), and 100% (FSM100), respectively. Cumulative gas production data were fitted to the France et al.^17^ nonlinear model using PROC NLIN (SAS 9.4). Model convergence was confirmed for all treatments. Goodness-of-fit: FSM0 R2 = 0.997, RMSE = 5.21; FSM25 R^2^ = 0.998, RMSE = 4.87; FSM50 R^2^ = 0.996, RMSE = 5.63; FSM75 R^2^ = 0.997, RMSE = 5.18; FSM100 R^2^ = 0.996, RMSE = 5.44 mL/g DM. Residual plots showed no systematic deviations.

Figure 1 illustrates the in vitro ruminal gas production (mL/g incubated DM) of diets containing different levels of FSM. The kinetics of total gas production were significantly affected by FSM inclusion (Table 4). The asymptotic gas volume (*b*) was highest in FSM25 (323.9 mL/g DM) and lowest in FSM100 (277.1 mL/g DM), representing a 7.8% decrease compared to the control (FSM0, 300.7 mL/g DM) (quadratic *P* = 0.014). The fractional rate of gas production (*c*) was significantly lower in FSM100 (0.035 /h) compared with FSM25 (0.045 /h) (quadratic *P* = 0.030). Lag time increased linearly with increasing FSM levels from 1.20 h (FSM0) to 1.61 h (FSM100), corresponding to a 34% increase (linear *P* = 0.009, quadratic *P* = 0.023). Metabolizable energy showed a significant quadratic response (linear *P* = 0.005, quadratic *P* = 0.012), peaking at FSM25 (8.72 MJ/kg DM) relative to FSM0 (7.93 MJ/kg DM) and then declining progressively, with FSM100 recording the lowest value (7.19 MJ/kg DM), a 17.6% reduction compared with the FSM25 peak. The PF_48_ and GY_24_ were not significantly affected by FSM inclusion.

**Table 4:** Effects of replacing soybean meal with graded levels of fenugreek seed meal on in vitro ruminal gas production kinetics, metabolizable energy, partitioning factor, and gas yield at 24 h.

	Diet^1^					SEM	*P* value		
	FSM0	FSM25	FSM50	FSM75	FSM100		Diet	Linear	Quadratic
*b*	300.7b	323.9a	283.7bc	282.9bc	277.1c	7.20	0.005	0.208	0.014
*c*	0.039ab	0.045a	0.038ab	0.037ab	0.035b	0.0025	0.016	0.896	0.030
*Lag*	1.20c	1.38b	1.46b	1.50ab	1.61a	0.046	0.004	0.009	0.023
ME	7.93b	8.72a	7.57bc	7.47bc	7.19c	0.236	0.007	0.005	0.012
PF_48_	2.15	2.07	2.26	2.28	2.28	0.105	0.552	0.478	0.641
GY_24_	336.0	360.6	317.2	311.9	311.4	18.06	0.302	0.451	0.423

Means in the same row with different letters differ, *P* < 0.05. P-value is the observed significance level of the F-test for diet; SEM=standard error of the mean.

^1^Fenugreek seed meal (FSM) was included at 4.3%, 8.6%, 12.9% and 17.3% to replace soybean meal at 25% (FSM25 diet), 50% (FSM50 diet), 75% (FSM75 diet) or 100% (FSM100 diet), respectively.

*b*, asymptotic gas production (mL/g DM); *c*, fractional rate of gas production (h^− 1^); *Lag*, lag time before the start of gas production (h); ME, metabolizable energy (MJ/kg DM); PF_48_, partitioning factor at 48 h (mg truly degraded substrate per mL gas produced); GY_24_, gas yield at 24 h (mL gas per g DM).

### Nutrient degradability and ruminal fermentation

All of *d*DM, *d*NDF and *d*ADF were significantly affected by FSM inclusion (Table 5). *d*DM decreased from 548 g/kg DM (FSM0) to 502 g/kg DM (FSM100), an 8.4% reduction (linear *P* = 0.008, quadratic *P* = 0.037). Similarly, *d*NDF linearly decreased by 15.6% from 546 g/kg DM in FSM0 to 461 g/kg DM in FSM100 (linear *P* = 0.038). Moreover, *d*ADF decreased both linearly and quadratically, from 491 g/kg DM in FSM0 to 434 g/kg DM in FSM100 (linear *P* = 0.016, quadratic *P* = 0.025), an 11.6% reduction.

**Table 5:** Effects of replacing soybean meal with increasing levels of fenugreek seed meal on in vitro dry matter and fiber digestibility, ruminal pH, total and individual volatile fatty acids, and ammonia-N concentration.

	Diet^1^					SEM	*P* value		
	FSM0	FSM25	FSM50	FSM75	FSM100		Diet	Linear	Quadratic
*d*DM (g/kg DM)	548ab	589a	538bc	535bc	502c	14.5	0.019	0.008	0.037
*d*NDF (g/kg DM)	546ab	571a	522bc	496 cd	461d	14.1	0.002	0.038	0.426
*d*ADF (g/kg DM)	491b	569a	463bc	461bc	434c	14.6	0.002	0.016	0.025
Ruminal pH	6.70b	6.68b	6.70b	6.81a	6.89a	0.028	0.007	0.014	0.040
Total VFA (mmol/L)	27.0b	29.9a	26.7b	26.0b	22.5c	0.67	0.002	0.032	0.025
C_2_ (mmol/L)	13.4b	15.1a	13.2b	12.9b	10.8c	0.52	0.002	0.013	0.037
C_3_ (mmol/L)	9.26b	10.18a	9.15b	8.78b	7.65c	0.288	0.001	0.035	0.015
C_2_:C_3_ ratio	1.45	1.48	1.45	1.47	1.41	0.042	0.809	0.555	0.983
C_4_ (mmol/L)	4.37	4.63	4.27	4.34	4.01	0.325	0.756	0.848	0.926
NH_3_-N (mg/dL)	12.3	12.6	12.1	12.0	11.4	0.38	0.316	1.000	0.886

Means in the same row with different letters differ, *P* < 0.05. P-value is the observed significance level of the F-test for diet; SEM=standard error of the mean.

^1^Fenugreek seed meal (FSM) was included at 4.3%, 8.6%, 12.9% and 17.3% to replace soybean meal at 25% (FSM25 diet), 50% (FSM50 diet), 75% (FSM75 diet) or 100% (FSM100 diet), respectively.

*d*DM, dry matter degradability; *d*NDF, neutral detergent fiber degradability; *d*ADF, acid detergent fiber degradability; VFA, volatile fatty acids; C_2_, acetate; C_3_, propionate; C_2_:C_3_, acetate-to-propionate ratio; C_4_, butyrate; NH_3_–N, ammonia nitrogen.

Rumen pH increased from 6.70 in FSM0 to 6.89 in FSM100 (linear *P* = 0.014, quadratic *P* = 0.040). Total VFA concentration was highest in FSM25 (29.9 mmol/L) and lowest in FSM100 (22.5 mmol/L), representing a 16.7% reduction relative to FSM0 (linear *P* = 0.032, quadratic *P* = 0.025). Acetate concentration decreased by 19.4% (from 13.4 to 10.8 mmol/L; *P* = 0.002), and C_3_ decreased by 17.5% (from 9.26 to 7.65 mmol/L; linear *P* = 0.035, quadratic *P* = 0.015) at FSM100 compared to FSM0. The C_2_:C_3_ ratio, and concentrations of C_4_ and NH_3_-N were not significantly affected.

### Methane and carbon dioxide production

**Fig. 2:**
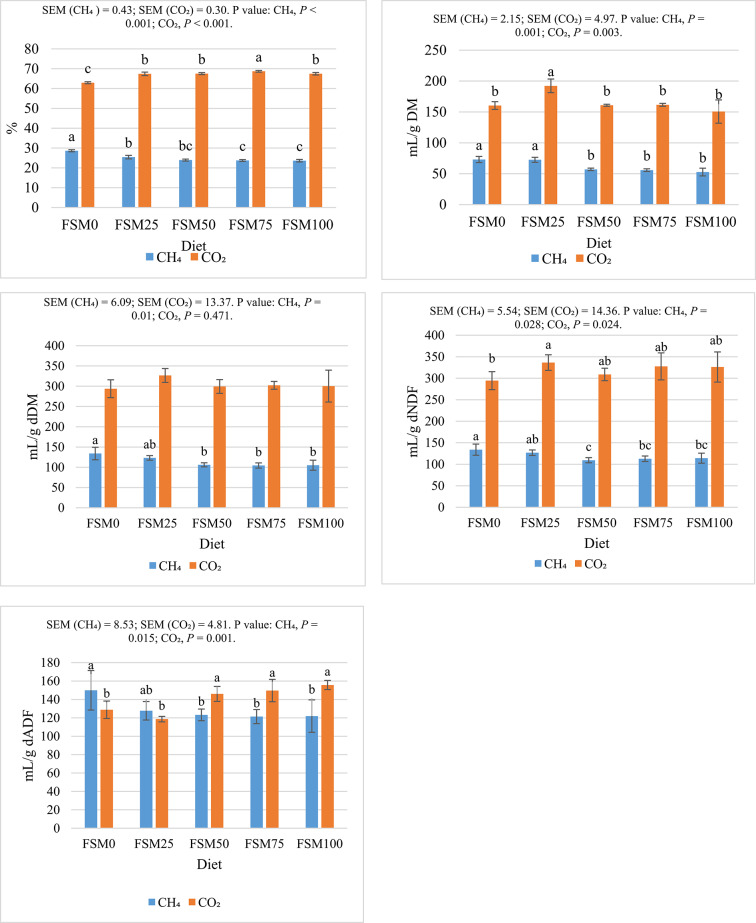
In vitro ruminal methane (CH_4_) and carbon dioxide (CO_2_) production, expressed as percentage of total gas, mL/g DM, mL/g digested DM (*d*DM), mL/g digested NDF (*d*NDF), or mL/g digested ADF (*d*ADF). Diets contained fenugreek seed meal (FSM) at 0, 4.3%, 8.6%, 12.9%, and 17.3% of DM, replacing soybean meal at 0% (FSM0), 25% (FSM25), 50% (FSM50), 75% (FSM75), and 100% (FSM100), respectively. Means for the same parameter with different superscripts differ, *P* < 0.05. P-value is the observed significance level of the F-test for diet; SEM=standard error of the mean.

Dietary inclusion of FSM significantly affected in vitro ruminal CH_4_ production across all expression bases (*P* < 0.05) (Fig. 2). Methane production, expressed as percentage of total gas, mL/g DM, mL/g degraded DM (i.e., *d*DM), mL/g degraded NDF (i.e., *d*NDF), and mL/g degraded ADF (i.e., *d*ADF), decreased progressively with increasing FSM level, with the lowest values observed at the highest inclusion rates (FSM75 and FSM100).

Similarly, CO_2_ production was influenced by diet (*P* < 0.05). Inclusion of FSM altered CO_2_ output expressed as percentage of total gas and per unit of DM, *d*NDF, and *d*ADF. Differences were not significant when CO_2_ was expressed per unit of *d*DM (*P* = 0.471).

## Discussion

This study demonstrated that replacing SBM with graded levels of FSM altered diet composition, ruminal fermentation dynamics, nutrient degradability, VFA profiles, and GHG outputs in a dose-dependent manner. Notably, a 25% replacement of SBM with FSM yielded the most favorable outcomes, suggesting a potential associative effect between the two meals that optimizes nutrient utilization and fermentation efficiency. By integrating changes in chemical composition, amino acid profiles, and polyphenols with in vitro fermentation responses, these findings provide mechanistic insight into how FSM influences rumen function and CH_4_ production.

### Diet composition

Incorporating FSM into the diets resulted in consistent shifts in chemical composition as SBM was progressively replaced. The experimental diets were not formulated to be isonitrogenous, isoenergetic, or isofibrous. Instead, the test ingredient was substituted on a weight-for-weight basis within the concentrate portion of the diet to simulate a practical feeding scenario and evaluate its effects under conditions representative of on-farm application. Increasing FSM inclusion elevated dietary fiber content while slightly reducing the proportion of readily fermentable carbohydrates. These compositional changes are likely to influence ruminal fermentation characteristics. Dietary CP declined from 132.5 to 122.5 g/kg DM and NDF increased from 330.3 to 334.8 g/kg DM across the five diets. The progressive reduction in CP and NSC and increase in fiber fractions (e.g., NDF) with higher FSM inclusion reflect the inherent compositional differences between FSM and SBM. FSM has lower CP but higher fiber than SBM, thus shifting the fermentable substrate toward a more fibrous profile. This altered nutrient matrix likely contributed to slower fermentation and lower gas production observed at the highest FSM levels (75–100% replacement), since readily fermentable carbohydrate availability is a strong determinant of microbial energy generation and gas output in vitro^20^. Variations in diet composition have been repeatedly linked to differences in gas kinetics and fermentation profiles, with high-fiber, low-soluble carbohydrate diets typically producing less total gas and slower fermentation rates^20,21^.

The comparative amino acid profiles of SBM and FSM highlight fundamental nutritional differences that are important for ruminant diets. Soybean meal has long been considered a high‑quality protein source due to its balanced profile of essential amino acids, particularly branched‑chain amino acids like leucine, isoleucine, and valine^22^. Soybean meal clearly exceeded FSM in all measured essential amino acids. This aligns with established data on soybean meal’s rich essential amino acid content, which supports microbial protein synthesis and overall animal growth^22^. Lysine, often considered the first limiting amino acid in ruminant diets, was also markedly higher in SBM (12.1 g/kg DM) than in FSM (7.9 g/kg DM). Adequate lysine supports muscle accretion and feed efficiency, especially in high‑producing animals^23^. While FSM does contribute lysine, its lower concentration suggests that direct replacement of SBM with FSM might require lysine balancing through supplementation or blending with other ingredients to meet dietary requirements.

Among nonessential amino acids, glutamic acid dominated both meals, but was higher in SBM (69.4 g/kg DM) compared with FSM (29.2 g/kg DM). Glutamic acid is central to rumen microbial metabolism and ammonia assimilation^24^. Reduced levels in FSM could influence the patterns of nitrogen utilization during in vitro fermentation, potentially resulting in different ammonia and microbial protein dynamics compared with SBM. Interestingly, arginine was relatively elevated in FSM (17.1 g/kg DM). While arginine is classified as nonessential in ruminants due to microbial synthesis, it plays roles in immunomodulation and nitric oxide production^25^. This distinct amino acid signature may impart unique physiological effects, though its practical significance in rumen‑based systems warrants targeted investigation.

The absence of cystine and proline in FSM is notable. Cystine contributes to protein structural integrity and sulfur amino acid pools^26^, and its absence could diminish the sulfur amino acid balance of FSM‑based diets unless supplemented via other feedstuffs or additives. Proline serves as a key substrate for collagen synthesis and intestinal metabolism, and its absence may reflect seed composition rather than analytical error, a pattern that has been observed in other legume seed meals with limited proline content^27^.

Taken together, the amino acid data indicate that FSM has a lower overall protein quality than SBM, particularly in essential amino acids such as lysine, leucine, and valine. These findings emphasize that FSM should not be viewed as a nutritionally equivalent replacement for SBM solely on the basis of crude protein concentration. Rather, its value as a feed ingredient reflects a balance between its protein contribution and its phytochemical characteristics, which may provide fermentation-modulating effects but may also necessitate amino acid balancing when used at high inclusion levels. At low replacement levels (e.g., 25%), the partial inclusion of FSM may complement SBM, allowing for an associative effect that supports efficient rumen microbial protein synthesis and maintains adequate amino acid supply to the host. In contrast, higher FSM inclusion reduces the overall dietary essential amino acid content, potentially limiting microbial growth and protein synthesis, which can negatively affect nutrient degradability and fermentation efficiency. These results highlight that the benefits of FSM are dose-dependent, with moderate replacement achieving a balance between bioactive phytochemicals and protein quality, while excessive replacement compromises amino acid availability and rumen function.

The stark differences in phenolic compound profile between FSM and SBM likely have implications for rumen fermentation and greenhouse gas production. FSM was particularly rich in flavan-3-ols (notably catechin, 977.5 µg/g) and hydrolyzable tannin–related compounds (ellagic acid, 170 µg/g; methyl gallate, 165.6 µg/g), compounds known for strong antioxidant activity^28^. Elevated phenolic content (2014.9 µg/g in FSM vs. 1453.3 µg/g in SBM) could influence microbial populations in the rumen, potentially suppressing methanogens or altering fermentation pathways^29^. Catechins, a class of flavan‑3‑ols found abundantly in tea and other plant sources, have been shown in vitro to modify fermentation patterns, reducing methane production through direct inhibitory effects on methanogenic archaea^30^. Although the exact ruminal effects of catechin at the concentrations observed in FSM have yet to be fully characterized, the high levels detected suggest potential biological relevance. Ellagic acid and related hydrolyzable tannins can bind to proteins and carbohydrates, affecting nutrient digestibility and microbial access^31,32^. These interactions may reduce proteolysis and ammonia release, potentially lowering nitrogen losses, but can also depress overall digestibility if concentrations are excessive^29^. The presence of other FSM phenolic constituents such as flavonols (rutin, 167.7 µg/g) and hydroxycinnamic acid derivatives (coumaric acid, 32.6 µg/g) further contributes to this chemical complexity. Rutin has been reported to exert selective antimicrobial activity against certain bacterial groups, while coumaric acid may influence microbial metabolism depending on ruminal conditions, although both effects are typically concentration-dependent and context-specific^33^.

By contrast, SBM exhibited higher levels of chlorogenic acid and vanillin. Chlorogenic acid has antioxidant properties but generally occurs at lower levels in legumes compared with other plant families^34^. Vanillin, a phenolic aldehyde, may exert mild antimicrobial effects but is not widely recognized for major impacts on rumen fermentation at typical dietary concentrations^35^. These differences point to a qualitatively distinct polyphenol landscape in each meal, with FSM potentially offering stronger radical‑scavenging and microbial modulatory capacity.

### Gas production and fermentation kinetics

In vitro fermentation kinetics revealed that low FSM inclusion (25%) increased asymptotic gas production and ME, while higher FSM levels (75–100%) slowed gas production and reduced energy availability. The initial increase in gas production at FSM25 may be attributed to a balanced nutrient profile that still provided sufficient fermentable substrates and amino acids^11^, while moderate polyphenol levels may have selectively suppressed less efficient microbial pathways^36^. However, at higher FSM inclusions, the combined effects of higher fiber, lower fermentable protein, and elevated bioactive compounds likely restricted microbial fermentation, as reflected by reduced asymptotic gas production and ME^37^.

The observed increase in lag time with higher FSM inclusion signals slower microbial adaptation to the substrate, a common feature when diets are rich in fiber and secondary metabolites that require more time for microbial colonization and enzymatic breakdown^20^. Additionally, slower gas production rates at high FSM levels align with reduced substrate degradability, as higher fiber and complex phenolic matrices are less readily fermented than diets high in soluble carbohydrates. Mousa et al.^11^ reported that replacing cottonseed meal with FSM at 50, 75 and 100% did not affect the total in vitro gas production.

### Degradability and rumen fermentation

The in vitro *d*DM and fiber degradability (*d*NDF and *d*ADF) declined with increasing FSM levels. The reduction in *d*DM from 548 to 502 g/kg DM (− 8.4%) and the more pronounced decline in *d*NDF (− 15.6%) and *d*ADF (− 11.6%) indicate that fiber digestion was particularly sensitive to high FSM inclusion. Two main mechanisms likely explain these responses. First, replacing conventional protein sources with FSM altered the chemical composition of the diet, increasing structural carbohydrates and reducing readily fermentable substrates. Diets with higher fiber fractions generally exhibit lower in vitro degradability because a greater proportion of the substrate is less accessible to rapid microbial colonization and enzymatic attack^38–40^. Second, FSM is rich in polyphenolic compounds. Polyphenols, including flavonoids and tannins, can bind to proteins and structural carbohydrates, forming complexes that reduce microbial attachment and enzymatic hydrolysis^40^. This protective effect may partially shield nutrients from ruminal degradation. While such interactions can decrease proteolysis and NH_3_ release, they may also limit overall digestibility when inclusion levels are high. Similar effects have been reported with polyphenol-rich diets in vitro, where digestibility decreases despite reductions in CH_4_^40^.

The increase in ruminal pH from 6.70 to 6.89 with higher FSM levels aligns closely with the observed reduction in total VFA concentration. Higher ruminal pH is physiologically consistent with lower acid production. When fermentation intensity declines, acid accumulation slows, resulting in a modest elevation in pH^41^. This pattern supports the interpretation that elevated FSM levels suppressed overall microbial activity rather than shifting fermentation toward excessive acidogenesis. Total VFA declined by 16.7% at FSM100 relative to the control, with the highest concentration detected at FSM25. Because VFAs are the primary end products of carbohydrate fermentation, their reduction reflects diminished fermentation extent^42^.

Both C_2_ and C_3_ concentrations decreased significantly at FSM100. Acetate is primarily associated with fiber fermentation, while propionate is largely derived from starch and soluble carbohydrate metabolism^43^. The parallel reduction in both suggests a generalized suppression of fermentative pathways rather than a selective shift between hydrogen-producing and hydrogen-utilizing routes. The absence of significant changes in the C_2_:C_3_ ratio indicates that fermentation balance between structural and NSC pathways was relatively maintained, despite the overall decline in activity. Similarly, C_4_ and NH_3_–N concentrations were not significantly affected, suggesting that protein deamination and butyrogenic pathways were comparatively stable across treatments^44^.

Polyphenols may also contribute to these VFA shifts through antimicrobial effects. Flavonoids and tannins have been shown to modulate rumen microbial populations, including fibrolytic bacteria and certain fermentative species, thereby reducing total VFA production at elevated inclusion levels^29,32^. Importantly, moderate levels often exert subtler effects^36^, which may explain why FSM25 maintained higher total VFA concentrations than FSM100.

### Methane and carbon dioxide production

The consistent decline in CH_4_ production across all expression bases demonstrates that FSM exerted a robust and biologically meaningful effect on ruminal methanogenesis. Whether expressed as a proportion of total gas, per unit of incubated DM, or relative to degraded DM, NDF, or ADF, CH_4_ output decreased progressively as FSM inclusion increased, with the lowest values recorded at FSM75 and FSM100. Expressing CH_4_ relative to degraded substrates is particularly informative, as it indicates that the reduction was not merely a consequence of lower digestibility at high inclusion levels. However, because the C_2_:C_3_ ratio was not significantly affected across treatments and H_2_ was not directly measured, a definitive conclusion about a shift in fermentation stoichiometry or hydrogen partitioning cannot be made from the present data. The reduction in CH_4_ expressed per unit of *d*DM, *d*NDF, and *d*ADF may reflect reduced substrate availability for methanogens or possible inhibitory effects of FSM bioactive compounds on methanogenic pathways, but this interpretation requires microbial and hydrogen flux data for confirmation. It is important to distinguish between a reduction in CH4 that reflects genuinely more efficient fermentation (i.e., less CH_4_ per unit of degraded substrate) versus a reduction driven by overall suppression of rumen fermentation (i.e., less total fermentation and therefore less total CH4). At high FSM inclusion levels (75–100%), the concurrent reductions in *d*DM, *d*NDF, *d*ADF, total VFA, and gas production suggest that the latter mechanism, suppressed fermentation, was the predominant driver of CH_4_ reduction. However, CH_4_ expressed per unit of degraded DM was also significantly lower at FSM50 and FSM100, which provides some evidence for an efficiency-related component beyond substrate limitation alone. Both mechanisms likely operated simultaneously at high FSM levels, and their relative contributions cannot be fully resolved without hydrogen flux data and direct microbial analysis. No positive control for CH_4_ inhibition was included, limiting the ability to benchmark the magnitude of reduction against a reference inhibitor. Several interacting mechanisms likely explain this^1^. When hydrogen production declines, CH_4_ synthesis is inherently constrained. This substrate-driven limitation becomes more evident when CH_4_ is expressed per gram of degraded fiber, indicating reduced methanogenic efficiency rather than simply reduced fermentation.

Second, FSM contains substantial concentrations of polyphenols and saponins, both of which are known to influence rumen microbial ecology. Polyphenols may exert antimicrobial effects on methanogens and protozoa, as reported in other in vitro studies with polyphenol-rich plant extracts^29^, including studies in which fenugreek substrates produced less CH_4_ than conventional forages^45^. However, rumen microbial community composition, methanogen abundance, and protozoal counts were not measured in the present study, and this mechanistic interpretation therefore remains speculative. Saponins, widely reported in fenugreek seeds, can disrupt protozoal membranes, leading to partial defaunation and reduced interspecies hydrogen transfer between protozoa and methanogens^46^. Because protozoa host symbiotic methanogens, their suppression reduces CH_4_ output. Importantly, saponin content was not quantified in the FSM used in this study, which is a significant limitation of the present work. Published literature reports diosgenin-type saponin concentrations in fenugreek seeds of approximately 3 to 6 g/100 g DM, with substantial variation by cultivar and processing method, but the saponin content of the FSM used here was not measured. The mechanistic role of saponins in mediating the observed CH_4_ reductions therefore remains speculative^46^. Future studies should incorporate quantitative saponin analysis alongside polyphenol profiling to allow a more complete mechanistic interpretation. The dose-dependent nature of the response observed here aligns with previous evidence showing that increasing concentrations of plant secondary metabolites progressively depress methanogenesis in vitro^47^.

Carbon dioxide production was also significantly influenced by FSM inclusion across all expression bases. Carbon dioxide is a primary product of carbohydrate fermentation and serves as a substrate for hydrogenotrophic methanogenesis^48^. The observed alterations in CO_2_ output suggest that FSM modified overall fermentation intensity and carbon flow. At moderate inclusion levels, CO_2_ production may reflect efficient fermentation with redirected hydrogen away from methane^48^. However, at higher inclusion rates, reduced degradability likely contributed to lower total CO_2_ formation, consistent with diminished fermentation extent.

### Limitations

Several limitations of this study should be acknowledged: (1) The in vitro batch fermentation system does not replicate continuous rumen flow, passage rates, rumination, or post-ruminal digestion. (2) Gas composition was measured at the 48-h endpoint only; VFA, pH, and NH3-N were not sampled at intermediate time points. (3) Rumen microbial community composition, methanogen abundance, protozoal counts and enumeration, qPCR data, and 16S rRNA sequencing were not determined; all mechanistic attributions to polyphenol- or saponin-mediated suppression are therefore inferential. (4) Saponin content of the FSM batch was not quantified. (5) No positive control for CH_4_ inhibition was included. (6) The experimental diets were not isonitrogenous or isoenergetic. (7) F57 filter bags may have restricted protozoal access to the substrate. In vivo validation incorporating animal performance measurements, enteric greenhouse gas emissions, rumen microbial ecology analyses (including methanogen and protozoal populations), and economic assessments are required before practical recommendations regarding the replacement of soybean meal with fenugreek seed meal can be made.

## Conclusion

The present study demonstrates that fenugreek seed meal is best suited as a partial replacement for soybean meal (at up to 25% of soybean meal protein contribution), where it may enhance gas production and metabolizable energy without significantly compromising fiber degradability or volatile fatty acid production. At higher inclusion levels (50–100% replacement), the cumulative effects of increased dietary fiber, reduced non-structural carbohydrate content, and elevated polyphenol load suppress overall fermentation activity, reduce substrate degradability, and lower volatile fatty acid production, effects that are likely to translate to reduced productive performance in vivo. The observed reductions in methane and carbon dioxide emissions appear to be associated with a combination of substrate limitation, polyphenol-mediated modulation of microbial activity, and potential saponin-related effects. However, the mechanistic basis of these responses requires confirmation through in vivo trials incorporating microbial community profiling, hydrogen flux measurements, and quantitative characterization of bioactive compounds. The findings indicate that fenugreek seed meal may have practical value as a limited substitute for soybean meal under specific inclusion thresholds; however, its broader nutritional and environmental implications require validation in vivo before practical recommendations can be established.

## Data Availability

The datasets used and/or analyzed during the current study are available from the corresponding author on reasonable request.

## References

[1] Moss, A. R., Jouany, J. P. & Newbold, J. Methane production by ruminants: Its contribution to global warming. *Anim. Res.* **49**, 231–253 (2000).

[2] Ahmed, M. G., Elwakeel, E. A., El-Zarkouny, S. Z. & Al-Sagheer, A. A. Environmental impact of phytobiotic additives on greenhouse gas emission reduction, rumen fermentation manipulation, and performance in ruminants: an updated review. *Environ. Sci. Pollut. Res.* **31**, 37943–37962 (2024). 10.1007/s11356-024-33664-5 PMC11189335 38772996

[3] Pérez-Trejo, E. et al. Effect of replacing soybean meal (*Glycine max*) with sesame meal (*Sesamum indicum*) on productive traits, carcass characteristics, and gross profit margin in fattening lamb’s diets. *Trop. Anim. Health Prod.* **54**, 405 (2022). 36441434 10.1007/s11250-022-03406-1 PMC9705427

[4] Hassan, O. G. A., El-Garhy, G. M., Kholif, A. E. & Mousa, G. A. Wheat germ meal replaces cottonseed meal at different levels in diets of Ossimi lambs: Impact on growth performance, feed utilization and economic efficiency. *J. Anim. Physiol. Anim. Nutr. (Berl)*. **108**, 806–815 (2024). 38311826 10.1111/jpn.13935

[5] Ebeid, H. M., Kholif, A. E., Chrenkova, M. & Anele, U. Y. Ruminal fermentation kinetics of *Moringa oleifera* leaf and seed as protein feeds in dairy cow diets: in sacco degradability and protein and fiber fractions assessed by the CNCPS method. *Agroforest Syst.* **94**, 905–915 (2020).

[6] El-Tanany, R.R.A. et al. Impact of replacing soybean meal with sunflower meal, sesame meal, and black seed meal in diets of Barki lambs. *World’s Vet. J.* **11**, 670–677 (2021).

[7] Obeidat, B. S., Al-Khaza’leh, J. & Alqudah, A. M. Black cumin meal (*Nigella sativa*) as an alternative feed resource during the suckling period of Awassi ewes: Assessments of performance and health. *Anim. Feed Sci. Technol.* **306**, 115820 (2023).

[8] Zandi, P. et al. Fenugreek (*Trigonella foenum-graecum* L.): An important medicinal and aromatic crop. In *Active Ingredients from Aromatic and Medicinal Plants* (ed El-Shemy, H.) 10.5772/66506 (InTech, 2017).

[9] Zeng, X., Chen, Y., Li, W. & Liu, S. Application of fenugreek in ruminant feed: implications for methane emissions and productivity. *PeerJ* **12**, e16842 (2024). 38313019 10.7717/peerj.16842 PMC10838068

[10] Faisal, Z. et al. The multifaceted potential of fenugreek seeds: From health benefits to food and nanotechnology applications. *Food Sci. Nutr.* **12**, 2294–2310 (2024). 38628211 10.1002/fsn3.3959 PMC11016425

[11] Mousa, G. A., Kholif, A. E., Hassaan, N. A., El-Garhy, G. M. & Hassan, O. G. A. Effect of replacing cottonseed meal with fenugreek seed meal on feed intake, digestibility, growth, blood parameters and economics of fattening lambs. *Small Ruminant Res.* **236**, 107305 (2024).

[12] Jajic, I., Krstovic, S., Glamocic, D., Jakšic, S. & Abramovic, B. Validation of an HPLC method for the determination of amino acids in feed. *J. Serb. Chem. Soc.* **78**, 839–850 (2013).

[13] Goering, H. K. & Van Soest, P. J. *Forage Fiber Analyses. U.S. Department of Agriculture* (ARS-USDA, 1975).

[14] Fortina, R., Glorio Patrucco, S., Barbera, S. & Tassone, S. Rumen fluid from slaughtered animals: A standardized procedure for sampling, storage and use in digestibility trials. *Methods Protoc.* **5**, 59 (2022). 35893585 10.3390/mps5040059 PMC9326712

[15] AOAC. *Official Methods of Analysis of AOAC International* (AOAC International, 1997).

[16] Van Soest, P. J., Robertson, J. B. & Lewis, B. A. Methods for dietary fiber, neutral detergent fiber, and nonstarch polysaccharides in relation to animal nutrition. *J. Dairy. Sci.* **74**, 3583–3597 (1991). 1660498 10.3168/jds.S0022-0302(91)78551-2

[17] France, J., Dijkstra, J., Dhanoa, M. S., Lopez, S. & Bannink, A. Estimating the extent of degradation of ruminant feeds from a description of their gas production profiles observed in vitro: Derivation of models and other mathematical considerations. *Br. J. Nutr.* **83**, 143–150 (2000). 10743493 10.1017/s0007114500000180

[18] Blümmel, M., Steingaβ, H. & Becker, K. The relationship between in vitro gas production, in vitro microbial biomass yield and 15 N incorporation and its implications for the prediction of voluntary feed intake of roughages. *Br. J. Nutr.* **77**, 911–921 (1997). 9227188 10.1079/bjn19970089

[19] Menke, K. H. et al. The estimation of the digestibility and metabolizable energy content of ruminant feedingstuffs from the gas production when they are incubated with rumen liquor in vitro. *J. Agric. Sci.* **93**, 217–222 (1979).

[20] Morsy, T. A., Gouda, G. A. & Kholif, A. E. In vitro fermentation and production of methane and carbon dioxide from rations containing *Moringa oleifera* leave silage as a replacement of soybean meal: in vitro assessment. *Environ. Sci. Pollut. Res.* **29**, 69743–69752 (2022). 10.1007/s11356-022-20622-2 PMC9512743 35570255

[21] Baffa, D. F. et al. Evaluation of associative effects of in vitro gas production and fermentation profile caused by variation in ruminant diet constituents. *Methane* **2**, 344–360 (2023).

[22] NRC. *Nutrient Requirements of Dairy Cattle* (National Academies, 2001). 10.17226/9825

[23] Seleem, M. S. et al. Overview of encapsulated lysine and methionine and their impacts on transition cow performance and health. *Anim. Res. One Health*. **4**, 145–166 (2026).

[24] Wu, G., Bazer, F. W., Johnson, G. A., Satterfield, M. C. & Washburn, S. E. Metabolism and nutrition of L-glutamate and L-glutamine in ruminants. *Animals* **14**, 1788 (2024). 38929408 10.3390/ani14121788 PMC11201166

[25] Wu, G. Functional amino acids in growth, reproduction, and health. *Adv. Nutr.* **1**, 31–37 (2010). 22043449 10.3945/an.110.1008 PMC3042786

[26] Yin, J. et al. L-Cysteine metabolism and its nutritional implications. *Mol. Nutr. Food Res.* **60**, 134–146 (2016). 25929483 10.1002/mnfr.201500031

[27] Albaugh, V. L., Mukherjee, K. & Barbul, A. Proline precursors and collagen synthesis: Biochemical challenges of nutrient supplementation and wound healing. *J. Nutr.* **147**, 2011–2017 (2017). 28978679 10.3945/jn.117.256404 PMC5657141

[28] de Araújo, F. F., de Paulo Farias, D., Neri-Numa, I. A. & Pastore, G. M. Polyphenols and their applications: An approach in food chemistry and innovation potential. *Food Chem.* **338**, 127535 (2021). 32798817 10.1016/j.foodchem.2020.127535

[29] Kholif, A. E. & Olafadehan, O. A. Essential oils and phytogenic feed additives in ruminant diet: Chemistry, ruminal microbiota and fermentation, feed utilization and productive performance. *Phytochem. Rev.* **20**, 1087–1108 (2021).

[30] Kim, E. T. et al. Effects of flavonoid-rich plant extracts on in vitro ruminal methanogenesis, microbial populations and fermentation characteristics. *Asian-Australas J. Anim. Sci.* **28**, 530–537 (2015). 25656200 10.5713/ajas.14.0692 PMC4341102

[31] Manoni, M. et al. Gallic and ellagic acids differentially affect microbial community structures and methane emission when using a rumen simulation technique. *J. Agric. Food Chem.* **72**, 27163–27176 (2024). 39588639 10.1021/acs.jafc.4c06214 PMC11638960

[32] Kholif, A. E., Olafadehan, O. A. & Anele, U. Y. Tannins in ruminant feeding: Effects of tannin type and dosage on rumen fermentation, production performance, health, and sustainability. *Res. Vet. Sci.* **106181**10.1016/j.rvsc.2026.106181 (2026). 10.1016/j.rvsc.2026.106181 41990601

[33] Kim, D., Kuppusamy, P., Jung, J. S., Kim, K. H. & Choi, K. C. Microbial dynamics and in vitro degradation of plant secondary metabolites in Hanwoo steer rumen fluids. *Animals* **11**, 2350 (2021). 34438807 10.3390/ani11082350 PMC8388715

[34] Tarahi, M., Gharagozlou, M., Niakousari, M. & Hedayati, S. Protein–chlorogenic acid interactions: Mechanisms, characteristics, and potential food applications. *Antioxidants* **13**, 777 (2024). 39061846 10.3390/antiox13070777 PMC11273606

[35] Patra, A. K. & Yu, Z. Effects of vanillin, quillaja saponin, and essential oils on in vitro fermentation and protein-degrading microorganisms of the rumen. *Appl. Microbiol. Biotechnol.* **98**, 897–905 (2014). 23624710 10.1007/s00253-013-4930-x

[36] Forte, L. et al. From rumen to milk: Dietary polyphenols in dairy cows—A critical review. *Vet. Anim. Sci.* **31**, 100569 (2026). 41584691 10.1016/j.vas.2026.100569 PMC12828550

[37] Widiawati, Y. et al. Potential of seaweed (*Eucheuma cottonii*) supplementation to reduce methane production, improve fermentation, and modulate the microbial composition of forages and crop by-products during in vitro rumen fermentation. *Front. Vet. Sci.* **12**, 1607879 (2025). 41064259 10.3389/fvets.2025.1607879 PMC12501642

[38] Kholif, A. E., Gouda, G. A., Morsy, T. A. & Patra, A. K. The effects of replacement of berseem hay in total mixed rations with date palm leaves ensiled with malic or lactic acids at different levels on the nutritive value, ruminal in vitro biogas production and fermentation. *Biomass Convers. Biorefin*. **14**, 3763–3775 (2024).

[39] Kim, S. H. & Sung, H. G. Effects of different fiber substrates on in vitro rumen fermentation characteristics and rumen microbial community in Korean native goats and Hanwoo steers. *Fermentation* **8**, 611 (2022).

[40] Vastolo, A. et al. Assessment of the effect of agro-industrial by-products rich in polyphenols on in vitro fermentation and methane reduction in sheep. *Front. Vet. Sci.* **12**, 1530419 (2025). 39950086 10.3389/fvets.2025.1530419 PMC11821959

[41] Mao, J. & Wang, L. Rumen acidosis in ruminants: A review of the effects of high-concentrate diets and the potential modulatory role of rumen foam. *Front. Vet. Sci.* **12**, 1595615 (2025). 40496917 10.3389/fvets.2025.1595615 PMC12148896

[42] Brandao, V. L. N. & Faciola, A. P. Unveiling the relationships between diet composition and fermentation parameters response in dual-flow continuous culture system: a meta-analytical approach. *Transl. Anim. Sci.* **3**, 1064–1075 (2019). 32704870 10.1093/tas/txz019 PMC7200414

[43] Bergman, E. N. Energy contributions of volatile fatty acids from the gastrointestinal tract in various species. *Physiol. Rev.* **70**, 567–590 (1990). 2181501 10.1152/physrev.1990.70.2.567

[44] Gozho, G. N. & Mutsvangwa, T. Influence of carbohydrate source on ruminal fermentation characteristics, performance, and microbial protein synthesis in dairy cows. *J. Dairy. Sci.* **91**, 2726–2735 (2008). 18565931 10.3168/jds.2007-0809

[45] Niu, H. et al. In vitro ruminal fermentation of fenugreek (*Trigonella foenum-graecum* L.) produced less methane than that of alfalfa (*Medicago sativa*). *Anim. Biosci.* **34**, 584–593 (2021). 32777891 10.5713/ajas.20.0114 PMC7961276

[46] Kholif, A. E. A review of effect of saponins on ruminal fermentation, health and performance of ruminants. *Vet. Sci.* **10**, 450 (2023). 37505855 10.3390/vetsci10070450 PMC10385484

[47] Maredi Matabane, D., Ng’ambi, W., Mabelebele, J. & Gunya, M. B. & Grace Manyelo, T. The role of secondary metabolites on methane reduction in small ruminants. In *Latest Scientific Findings in Ruminant Nutrition—Research for Practical Implementation* (eds. Babinszky, L. & Payan-Carreira, R.) 1–24 (IntechOpen, 2024). 10.5772/intechopen.1005461

[48] Leahy, S. C. et al. Electron flow: key to mitigating ruminant methanogenesis. *Trends Microbiol.* **30**, 209–212 (2022). 35027237 10.1016/j.tim.2021.12.005

[49] MAFF. Energy Allowances and Feeding Systems for Ruminants. Technical Bulletin 33. (1975).

